# Fecal Microbiota Transplant for Hematologic and Oncologic Diseases: Principle and Practice

**DOI:** 10.3390/cancers14030691

**Published:** 2022-01-29

**Authors:** Maroun Bou Zerdan, Stephanie Niforatos, Sandy Nasr, Dayana Nasr, Mulham Ombada, Savio John, Dibyendu Dutta, Seah H. Lim

**Affiliations:** 1 Division of Medicine, State University of New York Upstate Medical University, Syracuse, NY 13210, USA; BouZerdM@Upstate.edu (M.B.Z.); NiforatS@Upstate.edu (S.N.); NasrS@Upstate.edu (S.N.); NasrD@Upstate.edu (D.N.); OmbadaM@Upstate.edu (M.O.); johns@Upstate.edu (S.J.); dibyendudutta12@gmail.com (D.D.); 2 Division of Gastroenterology, State University of New York Upstate Medical University, Syracuse, NY 13210, USA; 3 Division of Hematology and Oncology, State University of New York Upstate Medical University, Syracuse, NY 13210, USA

**Keywords:** fecal microbiota transplant, hematologic diseases, oncologic diseases, outcome, challenges

## Abstract

**Simple Summary:**

The transfer of a normal intestinal microbial community from healthy donors by way of their fecal material into patients with various diseases is an emerging therapeutic approach, particularly to treat patients with recurrent or refractory *C. difficile* infections (CDI). This approach, called fecal microbiota transplant (FMT), is increasingly being applied to patients with hematologic and oncologic diseases to treat recurrent CDI, modulate treatment-related complications, and improve cancer treatment outcome. In this review paper, we discussed the principles and methods of FMT. We examined the results obtained thus far from its use in hematologic and oncologic patients. We also propose novel uses for the therapeutic approach and appraised the challenges associated with its use, especially in this group of patients.

**Abstract:**

Understanding of the importance of the normal intestinal microbial community in regulating microbial homeostasis, host metabolism, adaptive immune responses, and gut barrier functions has opened up the possibility of manipulating the microbial composition to modulate the activity of various intestinal and systemic diseases using fecal microbiota transplant (FMT). It is therefore not surprising that use of FMT, especially for treating relapsed/refractory *Clostridioides difficile* infections (CDI), has increased over the last decade. Due to the complexity associated with and treatment for these diseases, patients with hematologic and oncologic diseases are particularly susceptible to complications related to altered intestinal microbial composition. Therefore, they are an ideal population for exploring FMT as a therapeutic approach. However, there are inherent factors presenting as obstacles for the use of FMT in these patients. In this review paper, we discussed the principles and biologic effects of FMT, examined the factors rendering patients with hematologic and oncologic conditions to increased risks for relapsed/refractory CDI, explored ongoing FMT studies, and proposed novel uses for FMT in these groups of patients. Finally, we also addressed the challenges of applying FMT to these groups of patients and proposed ways to overcome these challenges.

## 1. Introduction

The human intestinal tract is colonized by thousands of different microbial species. In the last two decades, various studies have established the importance of these microbial organisms in maintaining and facilitating human health and well-being. These commensal microbial communities play vital roles in regulating host metabolism, maintaining intestinal microbial homeostasis, and influencing the host’s adaptive immunity [1]. Consequently, it is not surprising that alterations in the normal microbial composition result in disease states. Therefore, it follows that restoring the intestinal microbial composition may treat disease states and ameliorate symptoms.

Many factors affect normal intestinal microbial composition [2]. The most common factor by far is medication, especially broad-spectrum antibiotics. In addition to removing the causative factors and waiting for the spontaneous normalization of the normal intestinal microbial community, probiotics and prebiotics may help with the recovery. However, the most rapid and effective method through which to restore the intestinal microbiome is through a fecal microbiota transplant (FMT) from donors with a normal intestinal microbial composition. FMT involves the instillation of stool that has been collected from a healthy donor and processed according to different institution-specific protocols into the intestinal tract of a patient with an altered intestinal microbiome. The term fecal microbiota refers to the complex array of microorganisms that live symbiotically within the intestinal tract of the host.

The concept of FMT is not new. It was first used in China in the form of a “yellow soup” in the fourth century to treat diarrhea [3]. There were also some reports about the consumption of fresh, warm camel feces by the Bedouins as a remedy for bacterial dysentery [4]. The first documented successful use of FMT was in 1958, when it was used to treat four patients affected by pseudomembranous colitis [5]. However, it was not until 1983 when the next case of successful use of FMT in a patient with *Clostridioides difficile* (*C. difficile*) infection (CDI) was reported [6].

FMT has since primarily been applied to patients with relapsed/refractory CDI. However, there has been increasing use of this therapeutic approach for other intestinal and systemic diseases, albeit on a research basis. Surveys in the United States and in Europe have indicated that the number of procedures being performed has climbed rapidly over the last few years [7,8]. Therefore, FMT is an emerging therapeutic approach with very broad potential applicability. Due to the complexity of the diseases and their treatment, patients with hematologic and oncologic diseases may be particularly suitable candidates for FMT. In this paper, we will discuss the principles and biologic effects of FMT, examine the factors rendering patients with hematologic and oncologic conditions to increased risks of relapsed/refractory CDI, explore the ongoing FMT studies, and propose novel uses of FMT in these groups of patients. Finally, we will address the challenges of applying FMT to these groups of patients and propose ways to overcome these challenges.

## 2. Steps and Biologic Effects of FMT

### 2.1. The Steps of FMT

**Figure 1:**
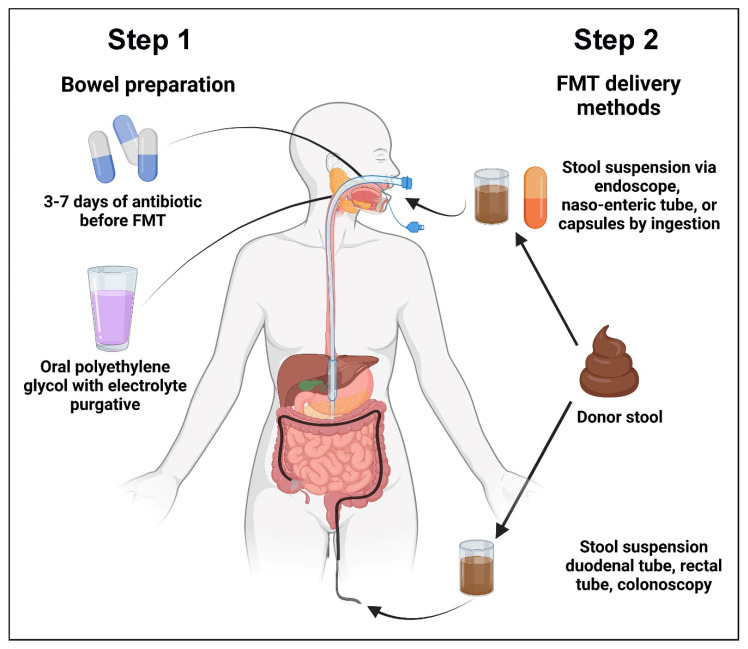
The two steps of fecal microbiota transplant. In Step 1, patients undergo bowel preparation with oral antibiotics followed by laxative. At least 24 h after the last dose of oral antibiotics, the patient will receive the donor fecal material via capsule, naso-enteral tubes, or upper or lower gastrointestinal endoscopy.

FMT can be divided into two steps (Figure 1): (1). bowel preparation and (2). decal material delivery. Step 1 of FMT involves bowel preparation using antibiotics to create the spatial niche for the transplanted microbes to populate and proliferate. The importance of this has been clearly demonstrated in a mouse model of FMT, in which pre-transplant antibiotic treatment facilitated more efficient engraftment compared to no bowel preparation or bowel preparation using a laxative [9]. Unlike in patients with CDI who usually have very restricted intestinal microbial diversity, bowel preparation with antibiotics may be even more important for successful FMT for non-CDI purposes. Based on these considerations, the European Consensus Conference on FMT recommends that patients with recurrent CDI should receive three days of either vancomycin or fidaxomicin before the FMT procedure [10], although we typically administer oral vancomycin for seven days prior to FMT in patients with active colitis, with the last dose being given 24 h before the procedure. The aim of the antibiotics is to decrease the abundance of *C. difficile* load and to create space for the establishment of the transplanted donor microbes. Routine administration of oral antibiotics in the absence of active colitis is generally not recommended due to concerns of diminished efficacy, especially in patients with diarrhea-predominant irritable bowel disease, in which antibiotic pretreatment has been shown to significantly reduce bacterial engraftment [11]. Bowel preparation with two to three liters of oral polyethylene glycol with electrolyte purgative is carried out on the day prior to FMT. Typically, 200–300 g of donor stool suspended in 200 to 300 mL of sterile normal saline is administered within ten minutes of the preparation of the stool mixture. The patients resume regular diet and medications two hours after the procedure. There is currently no consensus on the optimal protocol for FMT administration, and the protocol varies at each institution.

Up until 1989, fecal material was delivered by retention enemas. However, alternative methods were subsequently developed, including fecal infusion via duodenal tubes, rectal tubes, colonoscopy, and colonic transendoscopic enteral tubing [12,13]. Nowadays, enteral routes include the use of an endoscope, a naso-enteric tube, or capsules by ingestion. FMT for recurrent CDI is equally successful whether given via colonoscopy, nasogastric tube, or enemas administered at home [14]. A meta-analysis of four studies on the relative rate of CDI cure following oral FMT capsules compared to FMT delivered through colonoscopy was performed and did not find any differences in efficacy between the two methods. There were no reports of serious adverse effects that could be attributed to oral FMT capsules other than those associated with treatment failure. Oral FMT capsules are becoming more accessible and should be administered as per the protocol of the capsule manufacturer. One possible barrier to their use is that the number of capsules that has to be ingested for a full dose is frequently large and may lead to the gastrointestinal symptoms of nausea, vomiting, and bloating [15]. However, a larger meta-analysis involving 24 studies reported that FMT by lower gastrointestinal endoscopy was superior to all other delivery methods [16].

### 2.2. Biologic Consequences of FMT

**Figure 2:**
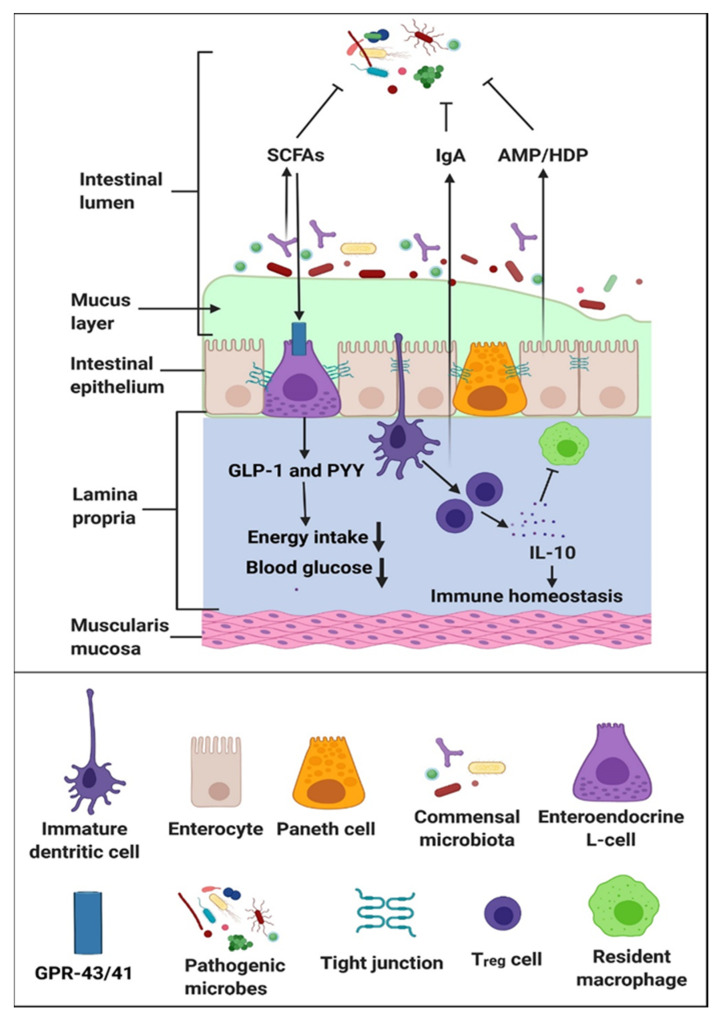
Fecal microbiota transplant restores intestinal microbial composition to modulate the adaptive immune responses, re-establish intestinal microbial homeostasis, and alter the host metabolism. Short chain fatty acids such as butyrate and propionate interact with G-protein coupled receptors GPR-43/41 on L cells to produce glucagon-like peptide 1 (GLP-1) and peptide YY (PYY), which contributes to reducing food intake and improving glucose metabolism [17].

Intestinal microbial communities regulate host metabolism, maintain intestinal microbial homeostasis, and modulate the host immune response (Figure 2) [17]. As a result, FMT re-equilibrates these functions in patients with the disease state due to intestinal dysbiosis. Unlike CDI in which the intestinal dysbiosis is clearly characterized by an overgrowth of toxigenic *C. difficile* [13], it remains unclear if the intestinal dysbiosis observed in other pathologic conditions is just an association rather than causation. If the relationship between the disease state and the intestinal microbial composition is merely one of association, restoring the normal intestinal microbial profile will not result in the improvement of the disease state and the amelioration of symptoms.

Outside the context of hematologic and oncologic conditions, FMT has been used to regulate host metabolism in both animal models of obesity [18,19] and in obese humans [20,21]. FMT from lean donors resulted in variable improvements in the insulin-sensitivity in obese recipients [22,23] and in patients with metabolic syndromes [22,23]. Improvement was associated with an increased abundance of butyrate-producing intestinal microbes.

FMT has also been used to re-establish normal intestinal microbial homeostasis. Currently, the most common indication for FMT is for relapsed/refractory CDI. FMT restores the diversity of the intestinal microbial compositions to create an ecologic competition between organisms to overcome and treat *C. difficile* overgrowth. Success rates of nearly 90% have been reported in most studies in patients with recurrent/refractory CDI (Table 1) [24,25,26,27,28]. Restoration of the normal intestinal microbial composition may also successfully eradicate colonization by multidrug resistant organisms such as extended-spectrum beta-lactamase-producing (ESBL) *Escherichia coli* (*E. coli*) [29], vancomycin-resistant *Enterococcus* (VRE) [30], and carbapenem-resistant *Enterobacteriaceae* (CRE) [30].

Since the intestinal microbial community modulates host immune responses, FMT has been applied to patients with inflammatory bowel disease. Randomized studies and non-randomized studies with a control arm have found higher clinical remission at eight weeks in patients with ulcerative colitis who were treated with FMT compared to the groups treated with placebo colonoscopic infusion [31]. To date, there has not been any published randomized clinical trial of FMT in Crohn’s disease. However, a meta-analysis of 11 case series and uncontrolled observational cohort studies found that slightly more than 50% of the patients achieved clinical remission [32]. Administration of a second FMT within 4 months of the initial FMT treatment maintained the clinical benefits of the first FMT treatment [33].

FMT has also been tried in other conditions, such as in human irritable bowel syndrome [34,35] and autism spectrum disorder [36], and in mice and humans for multiple sclerosis [37,38] and in mice for Parkinson’s disease [39]. In all of these disease states, the target for FMT is the gut–brain axis, which may be related to the breakdown of the gut barrier functions due to changes in intestinal metabolomics, such as the decrease in the production of short chain fatty acids caused by alterations in the normal intestinal microbial composition.

## 3. CDI in Patients with Hematologic and Oncologic Diseases

Patients with hematologic malignancies are particularly at risk for the development of CDI. CDI occurs in 7–14% of cases [40], and recurrent CDI (rCDI) occurs in 11–31% [41,42] of patients with hematologic malignancies such as acute leukemias, multiple myeloma, and Hodgkin’s and non-Hodgkin’s lymphoma. The incidence of CDI in patients with acute myeloid leukemia has been reported to be between 4.8 and 9%, and in those who undergo autologous hematopoietic stem cell transplantation (HSCT), a rate between 4.9 and 7.5% is observed; in those who undergo allogeneic HSCT, between a 14–30.4% incidence is observed in allogenic HSCT recipients [43,44]. The cumulative risks for developing peri-transplant CDI for those patients undergoing allogeneic HSCT who had CDI within 9 months of the transplant was reported to be nearly 40% [45]. Similarly, the incidence of CDI among those with solid tumors was also very high, reported to be between 10–20% [46]. CDI in these patients adds to the morbidities of the already debilitated physical state due to the underlying malignancies and may contribute to treatment-related mortality. CDI-related mortality in these patients is approximately 20% [47]. CDI is, therefore, a significant complication in patients who are receiving chemotherapy for malignant diseases.

### 3.1. Factors Predisposing Patients to CDI

**Figure 3:**
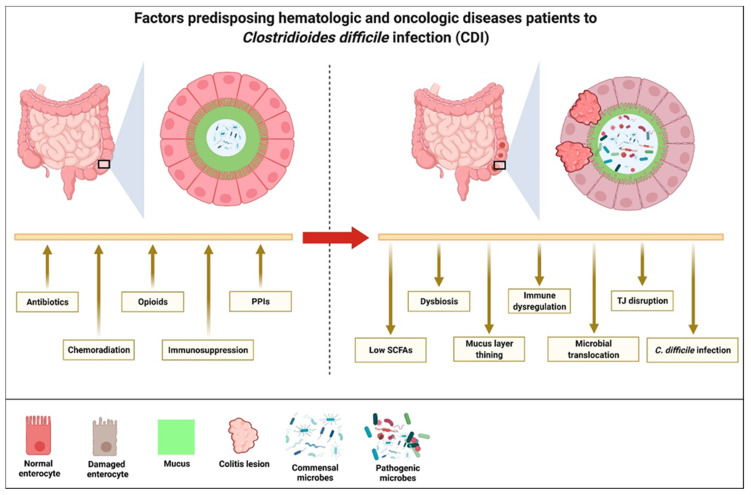
Patients with hematologic and oncologic diseases are more likely to develop *Clostridioides difficile* infection due to the frequent use of antibiotics, opioid analgesia and proton pump inhibitors, chemoradiation, and immunosuppressive agents. As a result, a change in the intestinal microbial composition and integrity of the epithelium results in the reduced production of short chain fatty acids, intestinal dysbiosis, thinning of the mucin layers, immune dysregulation, interruption to tight-junction formation, increased translocation of luminal microbes into the systemic circulation, and the development of *Clostridioides difficile* infections.

The mechanisms that are responsible for CDI pathogenesis in these groups of patients are multifactorial. In general, CDI risks are increased if there are changes to the normal commensal microbiota community (intestinal dysbiosis), innate intestinal immunity, or disruption to the integrity of the intestinal epithelial lining (Figure 3). By far, the biggest culprit contributing to the risks for CDI in these patients is the liberal use of broad-spectrum antibiotics that alter the intestinal microbial diversity and density, providing the opportunity for the colonization and proliferation of *C. difficile*, which is resistant to these antibiotics. Although the early initiation of broad-spectrum antibiotics reduces morbidity and mortality in patients who develop fever in the presence of chemoradiation-induced neutropenia [48], a retrospective study of 251 adult cancer patients found that despite patients having an absolute neutrophil count of more than 500/µL and 75% of the patients testing positive for a respiratory virus, 32% were still prescribed broad-spectrum antibiotics [49].

One of the first deterrence to *C. difficile* colonization in the intestine is the acidity of the gastric secretion. Both *C. difficile* spores and vegetative forms are inhibited by low gastric pH. It is therefore not surprising that the use of proton pump inhibitors (PPIs) is associated with an increased risk of CDI. A meta-analysis of 23 observational studies involving more than 300,000 patients found that PPI use was associated with a 65% increase in the incidence of CDI [50]. PPIs are often prescribed to hematologic and oncologic patients with severe thrombocytopenia and mucositis following chemoradiation therapy to reduce the risk of gastrointestinal bleeding. Therefore, PPIs increase the susceptibility of these patients to CDI.

The primary bile acids, chenodeoxycholic acid (CDCA) and cholic acid (CA), which make up 95% of the primary bile acids within the intestine foster *C. difficile* spore gemination to the vegetative cells within the ileum [51]. Medications that affect the transit time of these primary bile acids will favor the germination of the *C. difficile* spore to promote the colonization, proliferation, and induction of CDI. Opioids induce intestinal hypomotility that will increase the bile acid transit time. Opioids have also been found to induce intestinal dysbiosis [52]. The incidence of hospital-onset CDI among chronic opioid users is two times higher than that of the general hospital population [53]. Chronic opioid use to treat cancer-related pain therefore increases the risk of CDI in these patients by not only inducing intestinal dysbiosis but by also creating a condition that promotes the proliferation of *C. difficile*.

Patients with hematologic and oncologic diseases are rendered more susceptible to CDI because their innate host immunity is suppressed due to treatment [54]. This occurs due to the primary disease process or the one that is induced by the chemotherapeutic agents used to treat the diseases. Chemotherapeutic agents affect host immunity by their direct cytotoxic effects on the lymphocytes and by inducing neutropenia. In the setting of allogeneic HSCT, the use of immunosuppressive agents to prevent or treat graft-versus-host disease (GVHD) has also been found to increase host susceptibility to CDI [54].

Intestinal epithelial injury in the form of mucositis interacts bidirectionally with CDI. On the one hand, CDI induces mucosal damage, on the other hand, the presence of mucosal injury places the host an increased risk of CDI. Normal intestinal epithelium not only consists of enterocytes but also of supportive cells that include the goblet cells that are responsible for the production of mucin and Paneth cells that produce the antimicrobial peptide (AMP) [55]. Both intestinal mucin and AMP regulate the intestinal microbial community and density. Changes in the intestinal microbial community and density may not only result in alterations in the intestinal microbial metabolites such as in the short chain fatty acids (SCFAs) that play a major role in enterocyte health [56], but they may also create a niche favoring the colonization and proliferation of *C. difficile*. Damage to the normal intestinal epithelium, by chemotherapy or GVHD, will affect the integrity and functions of the goblet cells and Paneth cells and alter the production of mucin and AMP, respectively. Injury to the intestinal epithelium can also result in the release of damage-associated molecular patterns (DAMPs) that will also affect the intestinal microbial composition and density [57]. Cancer patients receiving chemotherapy that induces mucositis and patients with GVHD are there for at a higher risk for the development of CDI.

### 3.2. Use of FMT in Hematologic and Oncologic Patients outside Treatment of CDI

The immune regulatory effects of the intestinal microbial community have been exploited for treating acute GVHD following allogeneic HSCT. In total, the efficacy of FMT has been reported in 72 patients with corticosteroid-refractory acute GVHD (Table 2) [58,59,60,61,62,63,64,65]. Responses were observed in more than 50% of these groups of patients. More importantly, the procedures were all well tolerated, except for the development of lower gastrointestinal bleeding and hypoxemia in one patient and of bacteremia in two patients, although it was deemed unrelated to the FMT in all three cases. However, fatal donor-derived ESBL septicemia was reported in two patients who received FMT, one patient with hepatitis C infection in a clinical study of FMT for refractory hepatic encephalopathy, and another patient with therapy-related myelodysplastic syndrome in a study on the use of pre-emptive FMT following allogeneic HSCT [66]. The risks for such complications will be reduced as more stringent screen for the microbial composition in donors become more stringent.

Preclinical observations determined that the intestinal microbiota affected the response to immune checkpoint inhibitors (ICIs) [67]. Various retrospective studies also found that broad-spectrum antibiotics alter the intestinal microbial community and adversely impacted responses in cancer patients being treated with ICIs [68,69,70,71]. Based on these findings, two studies were performed on the use of FMT in a cohort of patients with immunotherapy-refractory malignant melanoma to determine whether the FMT could reverse the refractoriness to anti-Program Cell Death (PD) 1 immunotherapy. Three of the ten patients in one study restored the response to immunotherapy following FMT [72], and 6 of 15 in another study showed clinical benefits [73].

### 3.3. Ongoing FMT Studies in Patients with Hematologic and Oncologic Diseases

The initial successes observed with FMT in patients with hematologic and oncologic diseases have led to many clinical studies being currently ongoing in various institutions worldwide. Currently, there are nearly 40 studies registered with Clinicaltrials.gov. Table 3 shows the representative studies in the US and in Europe. These studies primarily evaluate the safety of FMT, the use of FMT to prevent and treat GVHD following allogeneic HSCT, improvement of ICI response, and the treatment of the complications that arise due to cancer therapy. It is expected that many of these studies will report their mature data on these outcomes within the next five years.

### 3.4. Harnessing the Potentials of FMT for Future Studies in Hematologic and Oncologic Diseases

The potential range of functions of a balanced intestinal microbial composition is wide. This provides great opportunities to tap into these potentials. Thus far, FMT has primarily been employed to restore the normal microbial homeostasis to treat CDI and to exploit the immune regulatory effects to treat corticosteroid-refractory GVHD following allogeneic HSCT and to restore the treatment responsiveness in melanoma patients who developed refractoriness to immunotherapy. Based on the assumption that the host immune system may have already developed a tolerance to the intestinal microbiota, it may be possible to extend the immune regulatory mechanisms of FMT to induce immune tolerance and to reduce the risk of developing intestinal GVHD using a combined allogeneic HSCT and FMT from the same donor.

Various studies have implicated a breakdown in the intestinal barrier function being responsible for the pathology of certain diseases. The breakdown of the intestinal barrier occurs frequently in patients with hematologic and oncologic diseases due to the direct cytotoxic effects of chemotherapy on the enterocytes or indirect effects of chemotherapy in modifying the intestinal microbiome and interrupting with the formation of the paracellular tight junctions (TJs) [74]. This increases the risks for the translocation of luminal bacterial products into the systemic circulation to induce culture-negative fever and bacteria to elicit bacteremia and septicemia. Fortifying the gut barrier and restoring the mucosal integrity using keratinocyte growth factors resulted in a reduction in the incidence of culture-negative fever and documented bacteremia/septicemia following high-dose chemotherapy and HSCT [75]. The facet of a balanced intestinal microbial composition in maintaining the gut barrier function through the production of the SCFAs that fortify enterocyte health, and paracellular TJ development may therefore be tapped into for similar purposes.

Recent studies in sickle cell disease (SCD) in mice [76,77] and in humans [78,79] have highlighted the presence and the role of disrupted gut barrier functions in affecting the phenotypes of the disease. This has been associated with intestinal dysbiosis that is characterized by a lower abundance of *Alistipes* and *Pseudobutyrivirio* [80]. Manipulation of the intestinal microbial community with the antibiotic rifaximin that led to an increased abundance of *Akkermansia* [81] was associated with a reduced frequency of painful vaso-occlusive crisis [82], creating an opportunity to use FMT from non-sickle cell donors with or without ex vivo enrichment with *Akkermansia* or *Alistipes, which* may be explored in the future to change the disease course in SCD.

## 4. Challenges Facing FMT Use in Hematologic and Oncologic Patients

The risk of introducing new infections remains the biggest concern of applying FMT to patients with hematologic conditions and oncologic patients. This anxiety among treating physicians has been amplified following the report on the fatal ESBL *E. coli* septicemia in patients with myelodysplastic syndrome who received pre-emptive FMT [66]. The risks are obviously higher in these groups of patients who are often neutropenic and immunosuppressed and who have an intestinal barrier that is already compromised. Therefore, any use of FMT in this group of patients, even if being used for CDI treatment, should be carried out in tightly controlled well-designed clinical studies.

Another challenge that faces these patients is the risk for bowel perforation and gastrointestinal bleeding due to instrumentation during FMT in a background context of intestinal mucositis. The development of capsule-delivered FMT should reduce this risk.

The biggest challenge affecting the successful use of FMT in patients with hematologic and oncologic diseases is the persistence of the factors predisposing these patients to the conditions that need FMT. Patients being treated for CDI will likely still require the frequent use of broad-spectrum antibiotics throughout the course of their cancer treatments. The continued use of systemic antibiotics has been found to predict FMT failure [83]. Even after completing the courses of chemotherapy, these patients also remain in an immunosuppressed state that predisposes them to further risks for CDI. In patients treated for GVHD, the intestinal microbiome likely reverses back to a dysbiotic state a few months after FMT since the alloreactivity persists in the background, and the use of immunosuppressive agents continues. Therefore, intermittent repeat FMT will be needed to maintain the restored intestinal microbial composition. The availability of capsule-delivered FMT may provide the solution, although it is still associated with the adverse events of diarrhea and abdominal discomfort/pain/cramping [84]. One could envisage the initial restoration of the intestinal microbial composition using a full FMT followed by daily/weekly maintenance of the microbiome using FMT capsules. Interestingly, a recent systematic review of the procedures performed over the last two decades found that FMT-related adverse events were the lowest when the colonic transendoscopic tubing method was used (6.33%) and the highest with gastroscopy (31.92%). The incidence of FMT-related adverse events was unexpectedly high with capsules (28.97%) [84], arguing against the safety of the capsule method in the treated patients, although how the capsule method compares with the other methods in patients with hematologic and oncologic diseases remains to be determined.

The problems associated with re-infection have been investigated in various studies. The incidence of failure and re-infection has been estimated to be around 14% [83]. Repeat FMT significantly reduces the failure rate in patients treated for CDI [16]. Patients who experience recurrence can, however, still be salvaged with bezlotoxumab [85].

## 5. Concluding Remarks

FMT is an emerging therapeutic approach that has an enormous number of potential applications. However, concerns remain among treating physicians on its use in patients with hematologic and oncologic diseases due to concerns related to introducing infections. A further barrier preventing the successful use of FMT in these groups of patients is the persistence of the factors predisposing the patients to the conditions needing FMT. Future work will focus on methods to overcome these obstacles. Until the indications are well-established, FMT in patients with hematologic and oncologic diseases should only be performed in closely monitored clinical trials.

## Figures and Tables

**Table 1:** Results of selected studies on the use of FMT for relapsed/refractory CDI.

Reference	Data Source	Number of Patients (n)	Outcome	Adverse Events
Youngster et al. [24]	A prospective study	180 using oral frozen capsules	CDI resolved in 82% of patients after a single treatment, rising to a 91% cure rate with two treatments.	Three cases of Grade 2 or above adverse reactions deemed related to the FMT were reported: One transient high fever, two new endoscopic diagnoses of ulcerative colitis.
Furuya-Kanamori et al. [25]	A collaborative analysis of patient data from 14 studies	305 (207 by lower and 98 by upper gastrointestinal route)	Risk of clinical failure was 5.6% and 17.9% in those treated by upper gastrointestinal route, and 4.9% and 8.5% in those treated by lower gastrointestinal route at Day 30 and 90, respectively.	Not reported.
Liu et al. [26]	Single center retrospective data	25 procedures (via feeding tube (n = 11), upper gastrointestinal endoscopy (n = 8), or colonoscopy (n = 6) in 24 patients)	Symptoms resolved in 21 of 24 patients (87.5%). Three patients who did not respond underwent a second FMT and all three responded	No serious adverse reactions were attributed to FMT.
Ponte et al. [27]	Single center retrospective study	34 (via upper gastrointestinal endoscopy (n = 30) or colonoscopy (n = 4)	Cure after one FMT in 22/25 (88%) and after two or more FMT in another 2/25 (8%).	No serious adverse reactions were reported.
Kelly et al. [28]	FMT National Registry Data	222 had follow-up at 1 month and 123 at 6 months.	90% cure rate at 1 month and 96% cure rate at 6 months.	At 1 month, 1% had hospitalization for diarrhea and severe abdominal pain, felt probably related to FMT; at 6 months, 1% developed irritable bowel syndrome and 1% inflammatory bowel disease.

**Table 2:** Reported results of FMT for corticosteroid-refractory GVHD.

Reference	Data Source	Number of Patients (n)	Outcome	Adverse Events
Kakihana et al. [58]	Single center prospective study	4 (received a total of 7 FMT by nasogastric administration)	3 CR and 1 PR	1 case of lower gastrointestinal bleed and hypoxemia, may not be related to FMT
Spindelboeck et al. [59]	Retrospective case series	3 (received a total of 9 FMT by colonoscopy)	2 CR and 1 PR	None reported
Qi et al. [60]	Single center prospective study	8 (received a total of 12 FMT by nasogastric administration)	5 CR and 1 PR	None reported
Shouval et al. [61]	Single center prospective study	7 (received a total of 15 FMT by capsule administration)	2 CR	2 episodes of bacteremia, deemed unrelated to FMT
van Lier et al. [62]	Single center prospective study	15 (received a total of 15 FMT by nasoduodenal tube administration)	10 CR	None reported
Zhao et al. [63]	Single center open-label Phase I/II study	41 (23 assigned to FMT and 18 to control. FMT administered by nasojejunal or gastric tube)	Overall response rate of 82.6% (52.2% CR and 30.4% PR) in the FMT group and 39% (all PR) in the control group on Day 14 after FMT, and an overall response rate of 69.5% (56.5% CR and 13% PR) in the FMT group and 50% (16% CR and 34% PR) in the control group on Day 21 after FMT	No difference in the adverse events between the FMT group and the control group.
Goeser et al. [64]	Two-center retrospective study	11 (9 by capsule and 2 by nasojejunal tube administration)	Attenuation of stool volume and frequency was observed in all 11 patients	Abdominal pain occurred in 3 patients and vomiting in 1 patient
Mao et al. [65]	Case report	1 (received two cycles of FMT administered by capsules)	CR	None reported

**Table 3:** Clinical studies registered in Clinicaltrials.gov for hematologic and oncologic patients in the US and in Europe.

NCT#	Study	Primary Outcome Measurements	Number of Patients (n)
02928523	PreventiOn of DYSbioSis Complications With Autologous FMT in AML Patients (ODYSSEE)	Evaluation of efficacy in dysbiosis correction and multidrug resistant bacteria based on bacterial culture	20
03678493	A Study of FMT in Patients With AML Allo HSCT in Recipients	Efficacy of FMT in AML patients and allo-HSCT recipients in reducing the incidence of infections	120
04935684	Faecal Microbiota Transplantation After Allogeneic Stem Cell Transplantation (TMF-Allo)	GVHD and relapse-free survival rate after allogeneic hematopoietic stem cell transplantation	150
04269850	Fecal Microbiota Transplantation With Ruxolitinib and Steroids as an Upfront Treatment of Severe Acute Intestinal GVHD (JAK-FMT)	Overall survival	20
05094765	Fecal Microbiota Transplant (FMT) Capsule for Improving the Efficacy of GI-aGVHD	Overall survival and Grade 3 or above adverse events	15
02269150	Autologous Fecal Microbiota Transplantation (Auto-FMT) for Prophylaxis of Clostridium Difficile Infection in Recipients of Allogeneic Hematopoietic Stem Cell Transplantation	CDI up to one year after entry into study	59
03214289	Fecal Microbiota Transplantation for Steroid Resistant and Steroid Dependent Gut Acute Graft Versus Host Disease	Serious adverse events	4
02733744	Fecal Microbiota Transplantation After HSCT	Feasibility on the number of participants able to ingest 15 FMT capsules over a 2-day period	18
03359980	Treatment of Steroid Refractory Gastro-intestinal Acute GVHD afteR AllogeneiC HSCT With fEcal Microbiota tranSfer (HERACLES)	Efficacy of FMT in steroid refractory -gastro-intestinal acute GVHD at Day 28	24
03819803	Fecal Microbiota Transplantation in aGvHD After ASCT	Remission at Day 90	15
04038619	Fecal Microbiota Transplantation in Treating Immune-Checkpoint Inhibitor Induced-Diarrhea or Colitis in Genitourinary Cancer Patients	Tolerability and response	40
02770326	Safety of Stool Transplant for Patients With Difficult to Treat C. Difficile Infection	Incidence of CDI	10
04116775	Fecal Microbiota Transplant and Pembrolizumab for Men With Metastatic Castration Resistant Prostate Cancer.	Anticancer effect of FMT from responders to pembrolizumab to non-responders.	32
04040712	Fecal Microbiota Transplantation in Diarrhea Induced by Tyrosine-kinase Inhibitors	Resolution of diarrhea four weeks after FMT	20
03819296	Role of Gut Microbiome and Fecal Transplant on Medication-Induced GI Complications in Patients With Cancer	Differences in stool microbiome pattern and adverse events	800
04951583	Fecal Microbial Transplantation Non-Small Cell Lung Cancer and Melanoma (FMT-LUMINATE)	Overall response rate	70
04521075	A Phase Ib Trial to Evaluate the Safety and Efficacy of FMT and Nivolumab in Subjects With Metastatic or Inoperable Melanoma, MSI-H, dMMR or NSCLC	Overall response rate and adverse events	42
04163289	Preventing Toxicity in Renal Cancer Patients Treated With Immunotherapy Using Fecal Microbiota Transplantation (PERFORM)	Rate of immune-related colitis associated with ipilimumab/nivolumab treatment	20
04729322	Fecal Microbiota Transplant and Re-introduction of Anti-PD-1 Therapy (Pembrolizumab or Nivolumab) for the Treatment of Metastatic Colorectal Cancer in Anti-PD-1 Non-responders	Overall response rate	15
04924374	Microbiota Transplant in Advanced Lung Cancer Treated With Immunotherapy	Measurements of safety	20
03341143	Fecal Microbiota Transplant (FMT) in Melanoma Patients	Overall response rate	18
03353402	Fecal Microbiota Transplantation (FMT) in Metastatic Melanoma Patients Who Failed Immunotherapy	Rate of adverse events and engraftment	40
04988841	Assessing the Tolerance and Clinical Benefit of feCAl tranSplantation in patientS With melanOma (PICASSO)	Safety and tolerability	60
04577729	The IRMI-FMT Trial	Progression-free survival	60
